# Notes and News

**Published:** 1888-01-28

**Authors:** 


### NOTES AND NEWS.

COLLECTED AND COMMUNICATED.

ABERDEEN is the latest claimant to the honour of being the first town to establish a Hospital Sunday collection, having begun in 1764. The last collection, made on New Year's Day, amounted to £800.

MR. M. SINGER has purchased an acre of land in the town of Paignton, on which he purposes to erect a cottage hospital for the benefit of the town. With exceptional generosity, Mr. Singer purposes endowing the institution as well as building it, so that the townspeople will enjoy the benefit of the institution without any call on their charity.

A MEETING of the Council of the Hospital Sunday Fund was held at the Mansion House, on Monday, under the presidency, first, of the Rev. Professor Marks, and then of the Lord Mayor. The main business for discussion was the appointment of committees. The Distribution Committee was selected as follows: The Lord Mayor, Sir Sydney Waterlow, General Sir E. C. Hodge, G.C.B., Mr. J. D. Allcroft, Mr. Thomson Hankey, Mr. F. H. Norman, Dr. Sedgwick Saunders, and Mr. A. Willett. The General Purposes Committee of last year were reappointed, Mr. H. C. Burdett and Mr. C. Cheston being elected to fill two vacancies. The honorary secretaries, Sir E. Hay Currie and Mr. R. B. Martin, and the secretary, Mr. H. N. Custance, were unanimously re-elected, and the chairman passed, on the part of the whole Council, a high and well deserved encomium on the services of the last mentioned officer. A further donation of £200 from Mr. E. A. Hankey was reported. The sobject of the contrast between the contributions received from South London churches, and the awards made to South London hospitals and dispensaries was referred to the General Purposes Committee to consider.

LEEDS stands in the forefront of the Hospital Saturday movement, and its workpeople are an example to the industrial class throughout the land. We recently reported their voluntary gift to the infirmary of two ambulances, and now we have great pleasure in recording their contributions during three successive years to the support of medical charities. In 1885 these amounted to £1,883; in 1886, to £1,884 5*s.* 6*d.*; and in 1887, to no less than £3,692 4*s.* 3*d.*, or nearly double the sum of the preceding year. Of this, it is proposed to give the Infirmary £3,230 13*s.* 8*d.*, while the Women's Hospital will receive £184 17*s.* 2*d.*, and the Dispensary £276 18*s.* 4*d.* Meanwhile, the committee of the Workpeople's Hospital Fund are consulting together how they may raise the subscriptions to a still larger sum this year. The generosity of the Leeds people proves conclusively that our working men and women need only to have the needs and claims of hospitals set before them to become the most loyal, and, in proportion to their means, liberal supporters of them.

THE Queen's Hospital, Birmingham, has received £4,284, being about £50 more than last year, from the Hospital Sunday Fund.

IT has been decided to improve and enlarge the Bristol Eye Hospital, to fit it for the reception of eighteen in-patients, and a daily attendance of 150 out-patients. The estimated cost of the improvement is £3,000.

THE seventy-first annual general meeting of the governors and committee of the Royal Ear Hospital, Soho, was held on the 10th inst. The institution continues to increase in usefulness and popularity. During the past year the number of patients treated—the largest in any year—was 2,654 out-patients and 56 in-patients, giving 8,343 attendances. The total expenditure for the year amounted to £722 3*s.* 3*d.*

AT the annual meeting of subscribers to the Manchester Institution for Diseases of the Ear it was stated that the number of patients admitted during last year was 1,239, a considerable increase on all previous years; but the subscriptions were little more than during the first year of the existence of the institution; and it was only by the utmost economy that the managers have been able to make the income meet the expenditure. The Chairman, Mr. G. Blair, in speaking of the good work done by the institution in a quiet way, said that he thought the subscription list was very small for a wealthy city like Manchester.

AT the last meeting of the governors of Leicester Infirmary, Mr. T. F. Johnson stated, on behalf of the Finance Committee, that on the Infirmary account there was a balance in hand of £211 16*s.* 5*d.*; on the medical library account, £31 5*s.* 9*d.*; and on the chaplain's fund account, £80 10*s.* 1*d.* The Hospital Sunday collections amounted last year to £1,409, and the workpeople's contributions to £1,950. One hundred and six convalescent patients had been sent to various homes, free of expense to the hospital. Of these the Rev. C. Rodwell (Kimcote) provided for 45, and the Rev. T. Jerwood (Little Bowden) for 8. Besides this, £40 was subscribed by various friends for the purpose of providing Christmas gifts for the patients; so it is evident that the people of the ancient borough of Leicester appreciate the work done by their infirmary, and support it heartily. The committee gave special thanks to the house surgeon, Dr. Okell, Mr. Sugden, and Miss Rogers, the matron, for the way in which they had performed their duties.

THE Committee of the Great Northern Central Hospital, Caledonian Road, are again sending out an appeal for subscriptions to the building fund of the new hospital. It is, perhaps, necessary to remind our readers of the circumstances of this hospital. Founded in 1856 at King's Cross, with a view to supplying the needs of the then extreme north of London, the district around has increased so rapidly, and is so densely populated, that it has far outgrown the resources of the hospital. The present building has been condemned by competent authorities; and, indeed, is in no way suitable for a hospital, being only a few private houses thrown into one, and dignified by that name. A freehold site has been purchased in Holloway Road, and the first portion of the new hospital, containing sixty beds, is to be opened shortly. The hospital, when completed, will hold one hundred and fifty beds, and will cost £45,000. Up to date the subscriptions received or promised amount only to £14,000, leaving the serious sum of £31,000 to be raised before the hospital can be opened free from debt. As at present urgent cases have to be refused daily for want of room, the new hospital is absolutely necessary, and it is much to be regretted that so excellent a scheme should be hindered by lack of funds.

The following vacancies are announced this week, the amount of salary in each case being given after the name of the institution :—

**Resident Medical Officers.**

Belgrave Hospital for Children House Surgeon —£90.
Birkenhead Borough Hospital. Senior House Surgeon.
Brighton, Hove, and Preston Dispensary. Two House Surgeons. —£140, with apartments.
Birmingham General Hospital. Two Assistant House Surgeons.
Royal National Hospital for Consumption, Ventnor. Assistant Resident Medical Officer.
Royal Surrey County Hospital, Guildford. House Surgeon.—£80, with board.

**Non-resident Medical Officers.**

Bristol Royal Infirmary. Dental Surgeon.
Downpatrick Union. Medical Officer, Killyleagh Dispensary.—£105, and fees
Durham Union. Medical Officer of Health —£100.
Forrest Hill Provident Dispensary. Medical Officer.

**Nurses.**

Nottingham and Notts Nursing Institution. Medical, Surgical, and Monthly.—£25 to £30, with uniform.
Royal Surrey County Hospital, Guildford. Head Nurse for women's wards.—£26 to £28, with uniform and laundress.
Sheffield Nurses' Home. Medical, Surgical, and Fever Address Lady Superintendent.
Glasgow Maternity Hospital. Nurses to train.—Fee £10.
Workhouse Infirmary Nursing Association, 6, Adam Street, Adelphi. Trained Nurses—several.
Liverpool Cancer Hospital, Myrtle Street. One. Apply Matron.
Hampstead Home Hospital and Nursing Institute. Trained Nurses for private staff.—£20, with uniform
The Birkdale Trained Nurses' Home, Southport. Two Trained Nurses, Protestant and teetotal.

**Probationers.**

Torbay Hospital, Torquay, Devon. Working Probationer. Apply Matron.
Worthing Infirmary Probationer.—No premium; no salary first year; all found.

Mr. G. Q. Roberts, of Hertford College, Oxford, an old Blue, has been elected secretary of the London Hospital, Whitechapel. Mr. Roberts has already done good work amongst the artizan class, and his appointment may largely supplement the sources of income upon which reliance has heretofore been placed for the maintenance of this useful hospital. What a grand thing it would be if the inhabitants of East London could be prevailed upon to combine to support the great institution in Whitechapel, as the artizans have done in Leeds and elsewhere, so that £20,000 a year might be forthcoming, as is quite possible, from this source alone.

At the annual meeting of the governors of Canterbury Dispensary special thanks were given to Messrs. George and Brian Rigden, the surgeons, for their services during the recent epidemic of measles. This came on very suddenly, about 300 cases being treated in the first six weeks. Owing to the severity of the weather, and the poverty of many of those attacked by the disease, there had been a good many deaths; but medical skill, aided by the kindness of Mrs. Holland, who not only gave food and coals to the poor, but also provided for nursing, is successfully coping with it.

The annual meeting of the Edinburgh Hospital and Dispensary for Women and Children was held on the 29th ult., in the Society of Arts Hall, 117, George Street. Mr. Hugh Rose presided, and there was a large attendance, wholly composed of ladies. Miss Brown read the report of the executive committee. The number of patients had increased month by month, but owing to the want of funds the committee regretted their inability to admit more patients into the hospital; 3,580 prescriptions had been issued, and 1,990 visits paid to patients. For the past year, ending 1st September last, the receipts had amounted to £273 18*s.* 1½*d.*, with an expenditure of £286 10*s.* 7½*d.*, leaving a balance of £12 12*s.* 6*d.* due to the treasurer. A new feature in the work of the institution was the issuing to patients of tickets at a nominal price, which entitled them to visits from the residen medical officer. During eight months of this year no less than 100 of these tickets had been applied for.

A French newspaper, *La Lanterne*, has, in imitation of *Truth*, commenced a fund for providing children in hospitals with toys. The fund now amounts to 2,653 francs, besides a large number of presents. M. Quantin, the head of a firm that publishes a large number of the most artistic children's books, has contributed 400 francs, which he has placed unconditionally at the disposal of the editor. A considerable number of children have offered their mites—that is, their centimes—to *La Lanterne*, with letters explaining their wishes in regard to the disposal of them.

Her Royal Highness the Princess Christian has consented to undertake the collection of funds in England towards the support of Lady Roberts's "Homes in the Hills for Soldiers' Nurses in India." The secretary is Captain Wilford N. Lloyd, R.A., Naval and Military Club, Piccadilly, W. Her Royal Highness has formed a committee of ladies to assist her, consisting of her Royal Highness Princess Christian, president; Mrs. Jeune, 37, Wimpole Street, W.; Mrs. Clifford Lloyd, 17, Elm Park Gardens, S.W.; Miss Sherston, Evercreech, Somerset; and Miss Emily Loch (Lady-in Waiting to her Royal Highness), The Cottage, Bishopsgate, Englefield Green, any of whom will gladly receive subscriptions.

At a meeting of the Sanitary Committee of Grimsby Town Council a discussion took place about recent proceedings in the small-pox hospital. It seems that the staff of nurses is so small that it is exceedingly difficult to exercise a complete supervision of the patients. The result of this has been that one patient, named Chatterton, was able to leave the hospital during the night unobserved—a proceeding which ended in his death. Dr. Westlake explained that it was difficult to get nurses for the hospital, and added that in his opinion Chatterton's death was not accelerated by his leaving the building. Finally, it was decided to ask Dr. Collie, of the Homerton Small-pox Hospital, to send three more nurses to Grimsby.

It is not generally known that St. Thomas's Hospital, in spite of the newness of its appearance, is, as a foundation, the oldest hospital in London except St. Bartholomew's. Its history can be traced as far back as 1200, when the original institution in Southwark was officially known as St. Thomas Overy, and popularly as "Becket's Spital," in memory of the murdered prelate. In 1571 the chief physician was a Mr. Bull, whose emoluments consisted of a salary of twenty marks (£13 6*s.* 8*d.*) per annum, and a "house in the close." This gentleman was officially inferior to the governor. His status was, however, better than that of the surgeon, who ranked officially after the shoemaker and before the barber. *Nous avons changé tout cela.*

It is deeply to be regretted that class-jealousy, trade-unionism, or any other outside consideration should interfere with the prosperity of a hospital, as it seems to have done in Bolton. The working men's Hospital Saturday collection for 1887 amounts to the handsome sum of £1,100, but this is a decrease of £20 compared with 1886; and two complaints which were brought before the Hospital Saturday Committee gave a clue to the deficiency. Some of the working-men had refused to subscribe because they thought "the masters" were in some way associated with the Infirmary, and others withdrew their support because an injured "knob-stick" had been treated there. It is to be hoped that the complainants will feel the justice of the remark made by the Chairman (Mr. John Berry), "that the Infirmary was the visible evidence of Bolton's charity, and that in helping its work they should lay aside all minor differences and meet on the platform of common humanity."

